# Lignocellulosic feedstocks: research progress and challenges in optimizing biomass quality and yield

**DOI:** 10.3389/fpls.2013.00474

**Published:** 2013-11-21

**Authors:** Maurice Bosch, Samuel P. Hazen

**Affiliations:** ^1^Institute of Biological, Environmental and Rural Sciences, Aberystwyth UniversityAberystwyth, UK; ^2^Biology Department, University of MassachusettsAmherst, MA, USA

**Keywords:** biofuels, biomass, cell wall, energy crops, feedstocks, grasses, lignocellulose, recalcitrance

## Dedicated energy crops and models

Lignocellulosic biomass derived from energy crops and agricultural residues is a promising renewable source for the production of transportation fuels and bio-based materials. Plants exhibiting C4 photosynthesis are amongst the most promising dedicated energy crops as they possess tremendous intrinsic efficiency in converting solar energy to biomass. Van der Weijde et al. (2013) provide an excellent overview of the potential of five C4 grasses from the Panicoideae clade (maize, *Miscanthus*, sorghum, sugarcane, and switchgrass) as lignocellulosic feedstock for the production of biofuels. The authors discuss yield potential, biomass quality and genetic improvement of dual-purpose food and energy cultivars and dedicated energy cultivars through plant breeding and also highlight several research needs. Perennial growth habit provides a number of environmental advantages over annuals as bioenergy crops, including the requirement of less fertilizer, reduced soil erosion, and even the potential for soil carbon sequestration. Schwartz and Amasino (2013) review the importance of the ability of perennial crops to recycle nitrogen, reducing the need for energy intensive N fertilizer, and the subsequent production of potent NO*x* greenhouse gases. An interesting focus is on the importance of photoperiodic flowering and dormancy in switchgrass and the relevance of these traits to N recycling and genetic variation that contributes to these dynamics. Wang et al. (2013a) review carbon partitioning in sugarcane, which has a source-sink system that creates high concentrations of easily extracted and economically valuable stem sucrose. Nonetheless, the majority of carbohydrate in sugarcane is lignocellulose. A detailed understanding of the molecular, and physiological processes underlying the partitioning of carbon assimilates will provide targets to manipulate the balance between sucrose and lignocellulosic biomass as carbon sinks. These include sugar transport and localization and the engineering of novel sugar sinks. This classic example of a possible dual-purpose crop can also be improved through breeding and genetic engineering of cell wall properties to further optimize biofuel production. Slavov et al. (2013) review the current knowledge about cell wall genetics, chemistry and structure in *Miscanthus*. This includes the prospects of developing detailed molecular genetic and biochemical models of pathways relevant to biomass conversion efficiency. The life history and genome complexities exhibited by the *Miscanthus* species in question dictate that genome wide association studies are a necessary approach toward the genetic dissection of these traits. Potential targets for biomass improvement include cell wall regulatory genes, intercellular trafficking, and microtubule organization. Opportunities exist to functionally test gene-trait associations for cell wall quality in this bioenergy crop, short-term progress toward understanding of the molecular underpinnings of cell wall quality traits in *Miscanthus* will be driven by research in model grasses. *Setaria viridis* is a rapid cycling C4 panicoid grass with several attributes that make it an excellent model for bioenergy grasses. Petti et al. (2013) describe the composition and saccharification dynamics of *S. viridis* aboveground biomass as similar to sorghum, maize, and switchgrass, confirming its potential as model species for panicoid translational genomics. Another grass proposed as a model for energy grasses, forage grasses and cereals is *Brachypodium distachyon*. Rancour et al. (2012) present chemical composition data of cell walls from distinct organs and developmental stages. Results indicate similar cell wall composition to those previously determined for a diverse set of C3 forage grasses and cereals, highlighting the usefulness of *B. distachyon* as a model for temperate grasses. As with *S. virdis*, the authors report some differences for particular wall traits.

## Cell wall quality and conversion

The plant cell wall is a heterogeneous mix of polymers that interact to assemble a complex and recalcitrant matrix. Cell wall quality with respect to biomass conversion is determined by the relative abundances of the different polymers, polymer substitutions and modifications and the interactions among polymers.

It is well established that lignin quantity and monolignol composition is a major contributor to the recalcitrance of cell wall industrial processing. Reducing lignin content and modifying its composition have become major strategies for bioenergy feedstock development. Sattler and Funnell-Harris (2013) review the impact of lignin modifications on pathogen susceptibility in a range of plants species. Surprisingly, data from existing literature indicates that reducing lignin content and altering its composition will not inevitably increase susceptibility to pathogens and in some instances it has increased resistance. Wang et al. (2013b) review the processes that follow monolignol biosynthesis, including their acetylation and glycosylation in the cytoplasm, transport to the apoplast, and deglycosylation, and polymerization into lignins. The authors point out that lignification is best understood at the level of biosynthesis with the goal of reducing lignin content. Increasing our understanding of the subsequent events will reveal important control points that can be used to develop novel strategies for improving plant biomass quality. Saathoff et al. (2013) discuss the roles of class III peroxidases in lignification, and biotic, and abiotic stress response. The authors provide an analysis of switchgrass class III peroxidases and predict their mechanistic role in defense to biotic stresses such as insect herbivory. Transcription factor activity is a major mechanism controlling the expression of genes involved in lignin biosynthesis and secondary cell wall formation in general. Cassan-Wang et al. (2013) describe a strategy to identify candidate transcriptional regulators of lignin biosynthesis in *Arabidopsis thaliana* based on mutant transcriptome analysis and sequence homology. Genetic analysis of the resulting candidate transcription factors revealed several novel mutants exhibiting altered lignin deposition, adding to the emerging complexity of the transcriptional networks regulating secondary cell wall formation.

Most of the fermentable sugar in lignocellulosic biomass crops is glucose and xylose in the form of cellulose and xylan polymers, respectively. Cellulose biosynthesis occurs at the plasma membrane by a complex that includes a number of Cellulose Synthase A catalytic subunits that extrude para-crystalline microfibrils. Brabham and Debolt (2013) review the use of chemical genetics as a tool to examine cellulose biosynthesis with screens to identify novel compounds that target relevant pathways. These new drugs will provide powerful tools for the detection of new molecular players in cell wall biosynthesis and in elucidating cell wall dynamics. Although some of the genes involved in the biosynthesis of the hemicellulosic xylan polysaccharide have been identified in *A. thaliana*, many of the biochemical steps of xylan synthesis remain unknown. Psyllium mucilage is overwhelmingly heteroxylan rich, presenting an opportunity to discover genes involved in xylan production. Jensen et al. (2013) provide an in-depth analysis of RNA-Sequencing to reveal overabundant transcripts in psyllium mucilaginous tissue. Results suggest that the biosynthesis and structure of mucilage xylan is somewhat distinct from secondary cell wall xylan. The diversity of glycosyl transferases identified in the mucilage might provide targets for future research in understanding and manipulating xylan biosynthesis.

A distinct feature of grass cell walls is the abundance of ferulic acid that is ester-linked to the hemicellulose glucuronoarabinoxylan. These ester-linked ferulates can form covalent linkages between polysaccharides and lignin and impede digestibility. Molinari et al. (2013) correlate the expression of BAHD genes, previously identified as candidates for the feruloylation of glucuronoarabinoxylan, with levels of bound ferulate and para-coumaric acid in the various *B. distachyon* tissues and developmental stages. Their findings warrant further studies to demonstrate a functional role of these genes in feruloylation. Matrix polysaccharides such as hemicelluloses and pectins in both grasses and eudicots are *O*-acetylated, affecting their interactions with other polymers and negatively affecting cell wall hydrolysis. Pawar et al. (2013) review the functional aspects of *O*-acetylation in trees and enzymes involved in both acetylation and de-acetylation. Given the high impact of acetylation on the physico-chemical properties of wood, these enzymes are of particular interest as they provide tools to manipulate feedstock quality. Pectin is a small fraction of plant secondary cell walls, yet these complex polysaccharides might have a significant impact on biomass yield and quality. Xiao and Anderson (2013) describe how pectin modification and cross-linking, largely determined by methyl esterification, affect growth rate and expansion and in turn influence overall biomass yield. Pectin modification can also influence secondary wall development and cell adhesion. The authors also discuss the value of pectin-rich feedstocks such as sugar beet as a source of fermentable sugars and other high-value products.

Biomass pretreatment is currently a fiscally prohibitive step in lignocellulose bioethanol production. Less recalcitrant genotypes can ameliorate this cost as well as positive interactions between genotypes and pretreatments. Serapiglia et al. (2013) measured sugar release by enzymatic hydrolysis following hot-water pretreatments from a range of shrub willow (*Salix* spp.) genotypes with differing biomass composition. Although relationships between cellulose and lignin content and sugar release were identified, results indicate that it is difficult to predict feedstock quality based on biomass composition. This suggests structural characteristics influence the effects of pretreatment and subsequent hydrolytic efficiency. While this collection of articles emphasizes bioconversion of lignocellulosic biomass to liquid fuels, it is important to emphasize the viability of thermochemical conversion for the production of both fuel and electricity. Tanger et al. (2013) review thermochemical conversion technologies and plant attributes that influence the efficiency of that process. The authors highlight the importance of biomass traits less relevant to bioconversion such as H:C and O:C ratio and mineral content. They also describe methods for high-throughput phenotyping of cell wall biomass that are distinct from traditional biochemical analysis of cell walls.

The sustainable production of energy, chemicals, materials, food and feed from plants is at the heart of a bio-based economy. Vanholme et al. (2013) present an insightful review in which they discuss the various requirements necessary to successfully develop a carbon-negative bio-based economy that can help mitigate climate change. The authors highlight that the viability of a bio-based economy depends on the integration of three pillars: green biotechnology for primary biomass production, white biotechnology to produce products from biomass and the thermochemical pillar for the conversion of residual biomass streams. Innovation, integration of both fundamental and applied research, and interaction between the pillars will be critical to generate a sustainable bio-based economy.

Lastly, we would like to thank all the authors, reviewers and the Frontiers editorial office for their contributions that resulted in, what we believe, a very interesting and relevant topical issue.
